# Effect of intestinal microbiota on growth rate of *Babylonia areolata*

**DOI:** 10.1371/journal.pone.0322985

**Published:** 2025-05-07

**Authors:** Wang Zhao, Xingmei Huang, Haipeng Qin, Zhengyi Fu, Rui Yang, Zhenghua Deng, Jinyong Zhu, Danli Wang, Zhongming Zheng

**Affiliations:** 1 School of Marine Sciences, Ningbo University, Ningbo, China; 2 Key Laboratory of Efficient Utilization and Processing of Marine Fishery Resources of Hainan Province, Sanya Tropical Fisheries Research Institute, Sanya, China; 3 South China Sea Fisheries Research Institute, Chinese Academy of Fishery Sciences, Guangzhou, China; 4 Hainan Engineering Research Center for Deep-Sea Aquaculture and Processing, Sanya, China; 5 International Joint Research Center for Conservation and Application of Fishery Resources in the South China Sea, Sanya, China; 6 Agro-Tech Extension Center of Guangdong Province, Guangzhou, China; South China Agricultural University, CHINA

## Abstract

To analyze the differences in the intestinal microbiota structure of different growth rates of *Babylonia areolata*, the sequencing of the V3-V4 regions of 16S rDNA from intestinal samples was performed. Evaluation of the richness of microbiota of the samples by calculating Shannon index, Simpson index and Chao1 index showed that the community diversity and richness of the intestinal microbiota of *B. areolata* changed for different growth rates. There are differences in the diversity of the intestinal microbiota in the fast growth rate (FG) and slow growth rate (SG) groups. A total of 315,294 reads of 16S rDNA were obtained from 6 samples, and 17 phyla were identified by RDP classifier. After data standardization, the dominant phyla from FG and SG were Firmicutes*,* Proteobacteria, and Bacteroidetes, 50.23%, 39.31% and 8.39% in the FG group, and 79.97%, 12.05% and 6.21% in the SG group, respectively. The distribution of the intestinal microbiota in the FG group is relatively uniform, but the *Mycoplasma* in the SG group is the dominant genus accounting for about 63%. At the genus level, compared to the SG group, the FG group exhibited a significant increase in the abundance of *Exiguobacterium*, *Vibrio*, and *Escherichia-Shigella*, while the abundance of *Mycoplasma*, *Citrobacter*, and *Phascolaretobacterum* was significantly reduced. These findings provide novel information for studying the differences in the intestinal microbiota of *B. areolata* with different growth rates, and a foundation for future research on intestinal bacterial factors that may affect the growth and cultivation of *B. areolata*.

## 1 Introduction

There are a large number of microorganisms in the intestinal tract of animals, which maintain a dynamic and homeostasis balance and play an important role in host digestion, nutrient absorption and immunity [1–3]. When the structure of intestinal microbiota is destroyed, the host is at increased risk of disease, which affects its health [4,5]. Studies have shown that multiple factors, such as heavy metals [6] and diseases [7], can affect the physiological activities of *Babylonia areolata* through the gut microbiota. *B. areolata* also known as *Babylon* snail or spotted *Babylon*, is an important economical marine gastropod mollusc that is intensively cultured in Hainan, Fujian, Guangdong Province of China and some Southeast Asia countries such as Tailand, Vietnam and so on [8,9]. As one of the main economic shellfish cultivated in China, *B. areolata* have great economic value. However, as an invertebrate, it lacks special immune system to resist the infection of various pathogens and mitigate the impact of environmental pressure [10–12]. The surface of the intestinal mucosa is the main binding site for host interactions with environmental microorganisms and external antigens [13]. In a few cases, the inherent microorganisms in the intestine can directly damage and penetrate the intestinal epithelium or induce tissue damage and inflammation to cause aquatic animal diseases [14]. Previous studies have established that environmental factors such as nitrite nitrogen, pH, and salinity exert significant impacts on the abundance and diversity of gut microbiota in *B. areolata* [15]. Furthermore, heavy metal exposure has been shown to disrupt both the structural composition of intestinal microbiota communities and the profile of antibiotic resistance genes (ARGs) in this species, elevating risks of microbial drug resistance and host susceptibility to diseases within the intestinal ecosystem [6]. Despite these critical findings, current knowledge regarding the gut microbiota of *B. areolata* remains fragmented, particularly in terms of taxon-function relationships, resilience mechanisms under environmental stressors, and host-microbe crosstalk. Consequently, systematic characterization of the intestinal microbiota structure and functional dynamics in *B. areolata* is imperative to advance our understanding of its ecophysiological adaptations and inform sustainable aquaculture practices.

With the development of the biology technologies, based on high-throughput sequencing have been developed and successfully applied to the analysis of complex intestinal bacterial ecosystems in various disciplines [16–19]. This technology has been extensively used in the detection of aquatic animal breeding environment and intestinal microbes [2,18].

At present, the detection and analysis of intestinal microbes in aquatic animals mainly focus on fish and a few shellfish. Studies have shown that the intestinal structure and function of intestinal microbes play an important role in aquatic animal growth and are closely related to physiological process such as nutrition metabolism, energy balance, immune defense and gastrointestinal development, which are essential to maintain the stability of internal environment [20–23]. By improving the aquaculture water environment, aquaculture conditions and bait formulas, the structure and function of the aquaculture environment and the intestinal microflora are adjusted to achieve the goal of favorable growth of aquatic products [24,25]. So far, there are few reports about the structure and function of the intestinal microbes of *B. areolata.* At the same time, there are few reports on the comparison of intestinal microbe structure and function. Therefore, the influence of the difference in the growth gate on the intestinal microbiota diversity of *B. areolata* was analyzed, in order to understand the changing law. It provides theoretical reference for the development of functional feed and regulation of intestinal microbe structure in the future, and also provides reference basis for formulating the best plan for different growth gate of *B. areolata* breeding and disease prevention and control.

## 2 Materials and methods

### 2.1 Experimental material

*B. areolata* (Total weight: 0.12 ± 0.27 g; Shell height: 0.41 ± 0.06 mm; Shell width: 0.22 ± 0.04 mm) were produced the same batch of *B. areolata* by a demonstration aquafarm, Wangning, China. All *B. areolata* were derived from a single parental cohort, with larvae originating from synchronized spawning events to ensure developmental uniformity and the parental stock was obtained from the same population. During the experimental period, the water quality parameters were measured daily and maintained at ammonia nitrogen < 0.1 mg/L, nitrite nitrogen < 0.02 mg/L, salinity 25–33, pH 7.8–8.2, and dissolved oxygen > 5.0 mg/L. The water temperature range was 25–32 °C and the illuminance range was 5,000–10,000 Lux.

### 2.2 Experimental method

The experiment was conducted in a concrete basin with sand layer self-cleaning leaking floor. The experiment adopted a basin of 5.74 × 2.87 m with a water depth of 1.0 m. The pond bottom was covered with coarse freshwater sand of approximately 4–6 cm thickness. Fresh *Trachurus japonicus* were fed at 17:00 every day, and residual baits were pulled out 2–3 h later. The range of daily water exchange was 100%-300%. Filtered natural seawater was used in this study.

**Fig 1:**
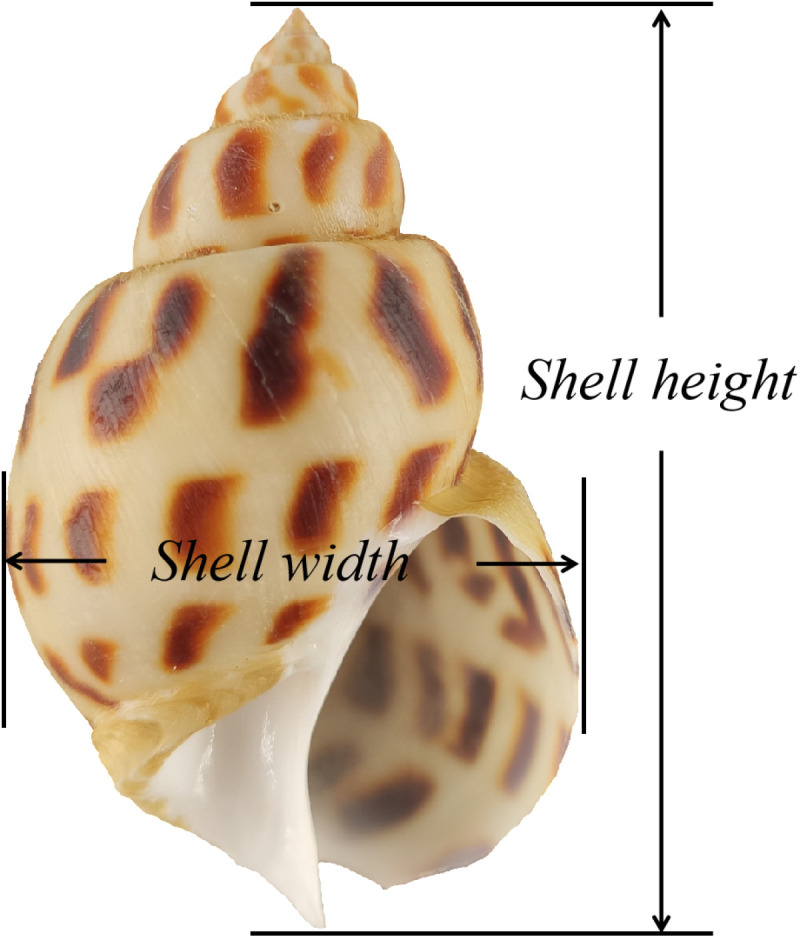
Shell height and shell width of juvenile *Babylonia areolata.*

180 days after rearing, about 16,000 juvenile *B. areolata* were graded according to shell height. In order to magnify the difference, the 10% *B. areolata* were separated of the minimum and maximum total weights, respectively, and defined them as “slow growth rate” (SG) and “fast growth rate” (FG). Three *B. areolata* were randomly sampled from each growth rate (The morphometric parameters of body weight, shell height, and shell width are presented in Table 1, while Fig 1 illustrates the morphological schematic delineating shell height and shell width measurements).

**Table 1:** Body weight, shell height and shell width sampled from all grades of *B. areolate.*

Size grade	Fast growth	Slow growth
Body weight (g)	11.16 ± 0.47	3.30 ± 0.29
Shell height (mm)	40.44 ± 0.37	25.95 ± 0.95
Shell width (mm)	24.85 ± 0.38	16.00 ± 0.41

### 2.3 Sample collection and processing

Clean the *B. areolata* thoroughly with sterile water and wipe the surfaces with 75% anhydrous ethanol. In the ultra-clean workbench, the intestinal tract of *B. areolata* was removed with sterilized anatomical tools and placed in a sterile 2 ml cryopreservation tube (Corning, New York, USA), which was stored at -80 °C after cold shock of liquid nitrogen.

### 2.4 DNA extraction

Total DNA was extracted from the intestinal contents using TIANamp Stool DNA Kits (Tiangen, Beijing, China), following the manufacturer’s instructions. DNA concentration was quantified using a NanoDrop 2000 (Thermo Scientific, Waltham, USA) by measuring absorbance at 260 nm (A260), while purity was evaluated through the A260/A280 and A260/230 ratios. DNA integrity was further confirmed by 1% agarose gel electrophoresis.

### 2.5 PCR amplifcation and 16S rDNA library construction

The V3–V4 hypervariable region of the bacterial 16S rDNA gene was PCR amplified using universal primers (F: 5’-CCTACGGRRBGCASCAGKVRVGAAT-3’ and R: 5’-GGACTACNVGGGTWTCTAATCC-3’). All PCR amplification were performed in triplicate at 25 μL reactions mixture containing: 2.5 μL of TransStart buffer, 2 μL of dNTPs mixture, 1 μL of each primer, 20 ng of template DNA. The thermal cycling program was performed as follows: initial denaturation at 94°C for 5 min, 30 cycles of denaturation at 94°C for 30 s, annealing at 55°C for 30 s, extension at 72°C for 30 s, and a final extension at 72°C for 10 min. The quality of amplified PCR products was checked by electrophoresis in 1.5% (w/v) agarose gel, then separated and purified with the Quick Gel Extraction Kit (Qiagen, Hilden, Germany). Purified PCR products were used for gene library construction and high-throughput sequencing.

### 2.6 Bioinformatics analysis

The concentration of DNA library was detected by Qubit 3.0 ﬂuorescent photometer, The DNA library was quantified to 10 nM and then loaded samples to Illumina MiSeq device (Illumina, San Diego, CA, USA) for sequencing according to the instruction. PE 250/300 was used for pairing with ends, picture analysis and base checks were performed by the MiSeq control software (MCS) attached to the MiSeq device. Paired-end reads were assigned to samples based on their unique barcode and truncated by cutting off the barcode and primer sequence. Pyrosequencing reads with ambiguous bases, quality score of Q ≥ 20, and reads shorter than 200 bp were removed. Raw data were merged using Flash (version v1.2.11) and filtered by QIIME (version v1.9.1). Uchime analysis was then performed to remove chimeric clusters from the sequencing data from each sample. Effective data were clustered at a 97% sequence identity into operational taxonomic units (OTUs) using UPARSE (version v7.0.1090) software, and taxonomic OTU assignments were accomplished by Ribosomal Database Project (RDP) Classifier. Rarefaction curves were analyzed with Mothur (version v.1.30.2). QIIME was used to calculate the bacterial alpha diversity index, including Shannon and Simpson (diversity), abundance-based coverage estimator (Ace) and Chao1 (richness), and coverage (the Good’s Coverage). Beta diversity was used as a comparative analysis of microbial communities in different samples. Heatmaps were generated with the R package.

### 2.7 Statistics and analysis

Data were analyzed using the SPSS 19.0 statistical software package (SPSS Inc., Chicago, USA). All the values are presented as means ± standard deviation (mean ± SD). Significant differences in the data were identified using one-way analyses of variance (ANOVAs). We considered *P* < 0.05 statistically significant.

## 3 Results

### 3.1 Microbial community richness and diversity

**Fig 2:**
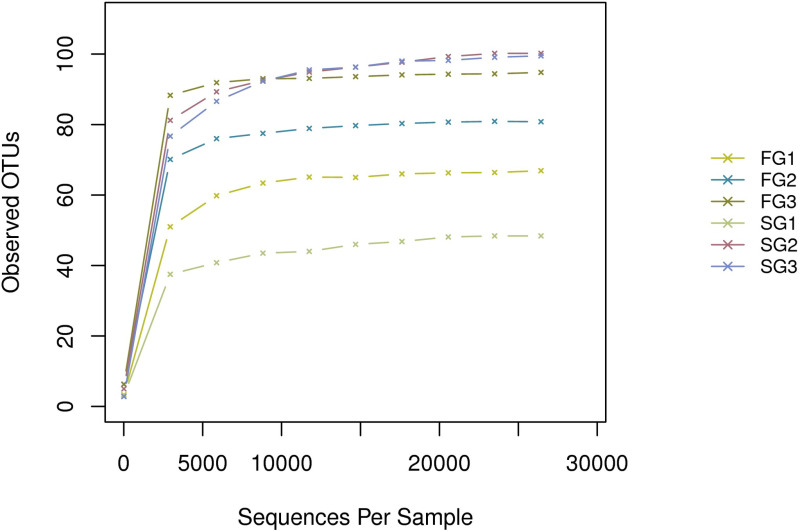
Rarefaction curve of the OTUs for six samples. FG1, FG2 and FG3 belong to fast growth group, SG1, SG2 and SG3 belong to slow growth group.

A total of 315,294 effective sequences were obtained from the total six samples after processing with the number of sequences ranging from 36,693–67,134 per sample. The average length of effective sequences was 459.66. The individual rarefaction curves showed a similar pattern of reaching plateau and the final trend is saturated (Fig 2). These results showed almost all microbial diversity has already been captured. The original 16S rRNA gene sequence data in this study were deposited at the National Center for Biotechnology Information by accession number PRJNA1194200 (https://www.ncbi.nlm.nih.gov/bioproject/PRJNA1194200)

**Fig 3:**
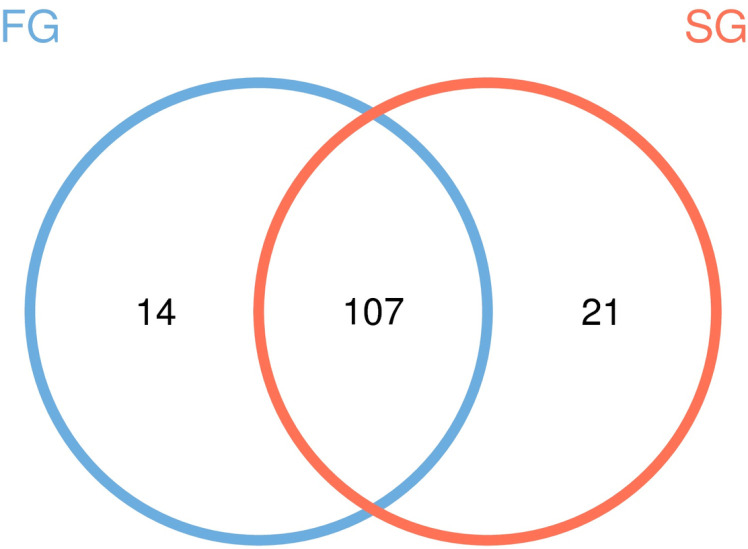
OTUs distribution in community level in venn diagram. FG, growth group rate; SG, slow growth rate.

The Venn figure could reflect the difference among groups FG and SG. According to the results of OTU cluster analysis, the common and unique OTU of intestinal microbiota of *B. areolata* with different qualities were analyzed. There were 121 and 128 OTUs in group FG and SG, and the percent of their common OUTs were 88.43% and 83.59%, respectively (Fig 3). The results show that there is a high similarity between the two groups.

**Fig 4:**
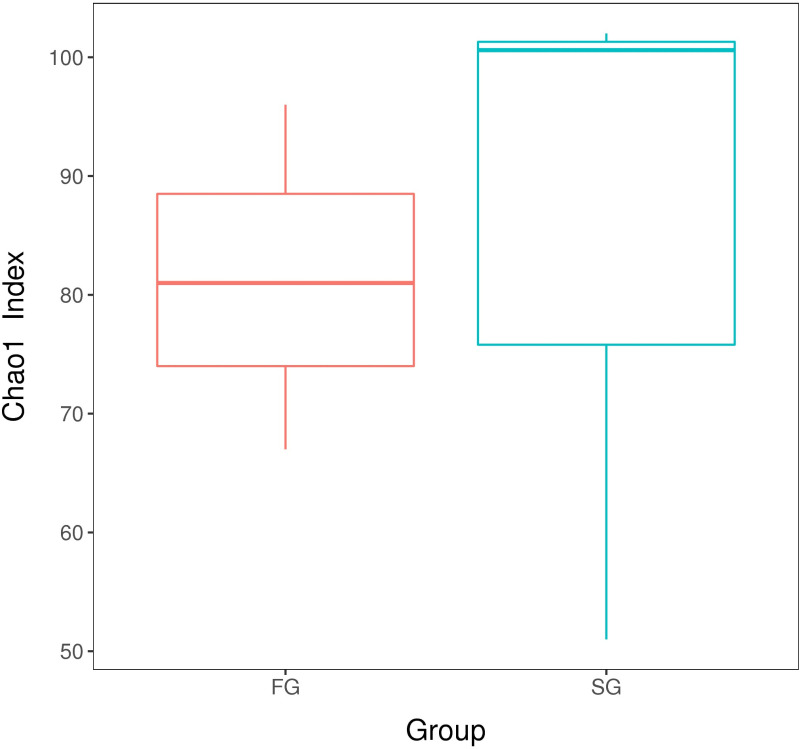
Comparative analysis of community diversity index. FG, growth group rate; SG, slow growth rate.

Chao1 index represent the community richness, the greater the index, the higher the community richness. The results showed that the highest Chao1 index in the two groups was SG(Fig 4), and the intestinal microbiota diversity in SG group was higher than that in SG group.

### 3.2 Taxonomic composition of bacterial communities

**Fig 5:**
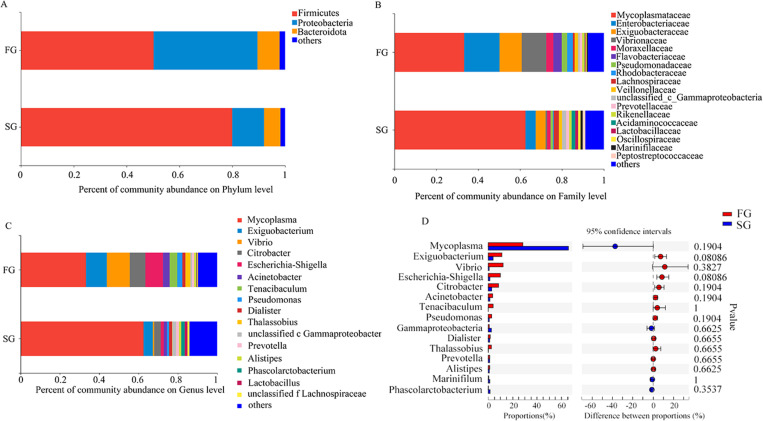
Compositions of the microbial communities at the phylum, Family and Genus level. A: Compositions of the microbial communities at the phylum level. Relative abundance of microbial groups (phylum level) in the intestinal samples of the FG and SG. B: Compositions of the microbial communities at the Family level. Relative abundance of microbial groups (Family level) in the intestinal samples of the FG and SG. C: Compositions of the microbial communities at the genus level. Relative abundance of microbial groups (genus level) in the intestinal samples of the FG and SG. D: Wilcoxon rank-sum test on Genus level with SG and FG groups.

The community structures of the samples on different classification levels were observed by a statistical analysis method in this study. A total of 17 phyla were identified by RDP classifier, which the relative abundance of their microbial communities was shown in Fig 5A. After data standardization, the dominant phyla from FG and SG were Proteobacteria, Firmicutes and Bacteroidetes. The test results of significance difference between FG and SG groups (Fig 5D) indicated that there was a great difference in the genus level of intestinal microorganisms between the two groups, but no significant difference was reached.

This analysis indicated that SG group was distinct from FG group. The dominant family in the SG groups was Mycoplasmataceae (62.56%), and their proportions was similar. In the FG group, the dominant family were Mycoplasmataceae (33.28%) and Enterobacteriaceae (16.92%), and their proportions was similar (Fig 5B).

At the genus level, the relative abundance of the top 30 microbial taxa was analyzed, revealing significant differences in intestinal microbiota composition between the FG and SG groups (Fig 5C). In the FG group, the dominant bacterial genus in *B. areolata* intestines were *Mycoplasma* (33.28%), *Exiguobacterium* (11.68%), *Vibrio* (10.57%), and *Escherichia-Shigella* (8.97%). In contrast, the SG group exhibited a distinct microbial profile dominated by *Mycoplasma* (62.58%), followed by *Exiguobacterium* (4.72%), *Citrobacter* (3.36%), and *Phascolarctobacterium* (1.74%). Notably, while the FG group showed a more evenly distributed microbial community (Shannon index: FG group: 3.365 vs. SG group: 2.412), the SG group displayed a pronounced dominance of *Mycoplasma*, which accounted for nearly twice the proportion observed in the FG group (62.58% vs. 33.28%, P < 0.01).

**Fig 6:**
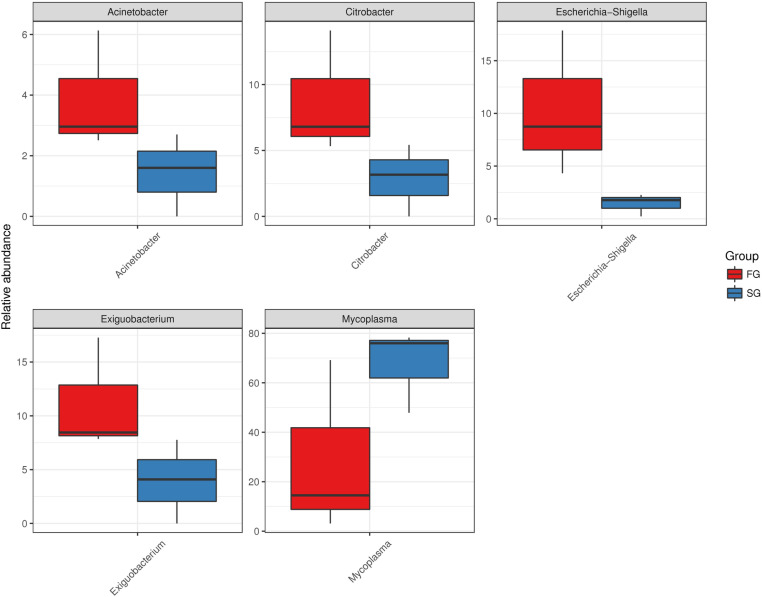
Comparison of the difference in microbial abundance between SG and FG group. FG, growth group rate; SG, slow growth rate.

Metastats software was used to analyze the significant difference between the two groups of samples and demonstrate the abundance distribution of the five strains (*Acinetobacter*, *Citrobacter*, *Escherichia-Shigella*, *Exiguobacterium*, *Mycoplasma*) with the largest difference in the two groups of samples (Fig 6). The results showed that *Escherichia-Shigella* in SG group and FG group were great different.

**Fig 7:**
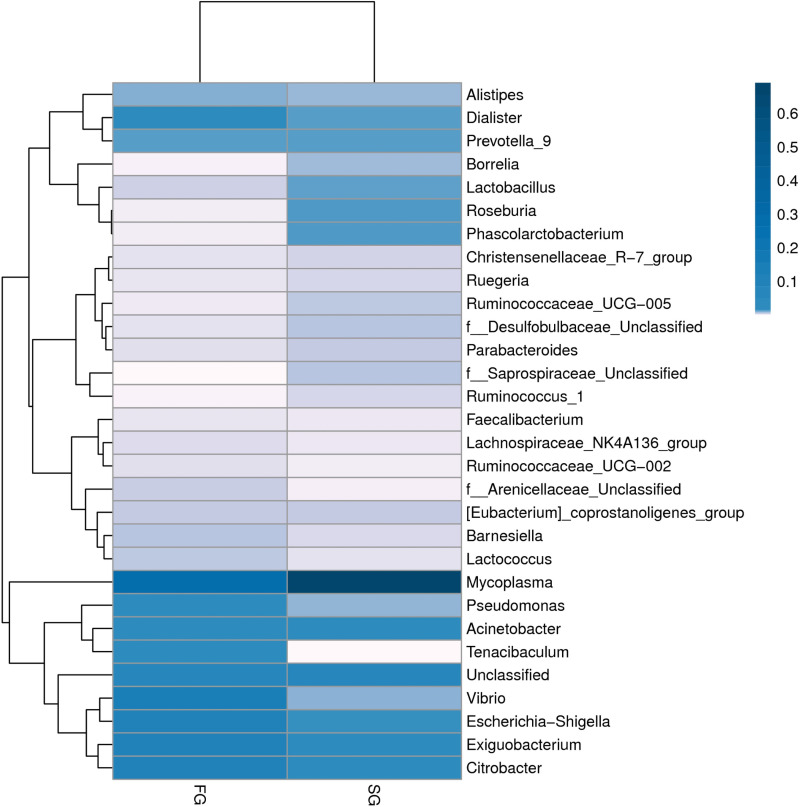
Heat-map of samples clustering of the FG and SG. FG, growth group rate; SG, slow growth rate.

A hierarchical clustering heat map analysis was performed at the genus level based on the top 30 most abundant microbial communities across two groups (Fig 7). The analysis revealed that the samples from SG have the highest diversity, and *Mycoplasma* is the bacteria genera of which the richness is 0.6.

## 4 Discussion

Intestinal microbiota play an important role in animal organism, and they are interdependent and interact with the host. When the structure of microbial community is destroyed, the host is at increased risk of disease, which affects its health [4,5,26]. Therefore, the use of 16S rDNA sequencing technology to study the influence of body weight on the intestinal microbiota community of *B. areolata* is of great significance for the development of its breeding technology and disease prevention and control. The results of this study showed that Proteobacteria, Firmicutes and Bacteroidetes for *B. areolata* were the dominant phyla, which was basically similar to the research results of other aquatic animals’ intestinal microbiota structure [27].

The results showed that the proportion of dominant phyla in the intestinal microbiota of FG and SG groups was different at the phyla level, but the species of dominant phyla were similar to those of other aquatic animals, such as the *Pinctada fucata martensii* [27], *Noble Scallop* [28]. Culture environment [29,30], metabolism [29], nutrient composition of feed [31], starvation [32], development [33,34] and other factors [35] can affect the composition of intestinal microorganisms. Under the condition of the same culture environment and feeding the same bait, the difference in total weight between the two groups was caused by the different proportion of Bacteroidetes. Bacteroidetes are closely related to the transformation of lipid, protein and other organic compounds [36,37], and play an important role in the maintenance of intestinal homeostasis [38], indicating that intestinal microbiota affects the transformation of organic compounds. How bacteroidetes affect the transformation of lipid, protein and other organic compounds needs further in-depth experiments.

At the genus level, *Mycoplasma* was one of the most abundant bacteria in the intestines of the two groups, and the abundance of SG group was more than 60%. Many *Mycoplasmas* are known to be pathogenic bacteria [39], and in some cases can infect humans and mammals and become intracellular pathogens. *Mycoplasmas* have been linked to chronic diseases in humans. Under appropriate environmental conditions, most species remain benign members of the host microbiome [40,41]. There was no disease or death in the SG group, indicating that *Mycoplasma* was a benign member of the intestinal microbiota of *B. areolata*. The abundance of *Mycoplasma* may be one of the indicators to judge whether the condition of low nutrition is in *B. areolata*. It is assumed that when SG group is suffering from chronic diseases or malnutrition, the increase of mycoplasma abundance may help the host to survive under the condition of low nutrition. This prediction needs further verification [42].

Research indicates that the growth rate of *B. areolata* is regulated by multifactorial mechanisms. Current evidence demonstrates that cultivation practices [43,44] and environmental parameters [45–47] influence its digestive processes and immune responses. Furthermore, nutritional disparities in diets and dietary supplements modulate growth performance by altering metabolic pathways and nutrient assimilation efficiency [48,49]. Furthermore, studies have revealed that the intestinal microbiota composition of aquatic animals exerts a direct regulatory influence on their growth performance to a certain extent [36,37], and the addition of probiotics in the breeding process can improve the digestion, nutrient absorption and immunity of aquatic animals to a certain extent [50]. At the same time, it is proved that the detection of intestinal microbiota structure of *B. areolata* has a certain positive significance in its breeding process [51,52]. This microbial-host interaction modulates nutrient metabolism, energy allocation, and physiological homeostasis, collectively contributing to organismal development. The distribution of intestinal microbiota in the FG group was relatively uniform, while *Mycoplasma* was dominant in the SG group. The observed inverse correlation between *Mycoplasma* abundance and *B. areolata* growth rate implies that accelerated host growth may correlate with diminished colonization of *Mycoplasma* within the gut microbiota. Current evidence suggests that gut-associated *Mycoplasma* often exhibits detrimental impacts on host fitness [53], including pathogenic potential [54–56]. However, contrasting studies report commensal *Mycoplasma* strains in the intestinal tract of *Atlantic salmon* (*Salmo salar*) that show no adverse health effects [57]. This discrepancy highlights the context-dependent role of *Mycoplasma*, which may vary across host species, microbial strains, and environmental conditions. Consequently, the functional significance of *Mycoplasma* in modulating growth performance of *B. areolata* remains unresolved and warrants further investigation through strain-specific genomic analysis and in vivo functional assays. The results of this study were analyzed in combination with relevant literature. To some extent, the species and distribution of intestinal microbiota affected the absorption and transformation of organic matter by *B. areolata*, thus affecting its growth and development [36–38,50].

With the deepening of the research on the intestinal microbiota, more and more evidence has shown that the intestinal microbiota plays an indispensable role in regulating host health and disease. It has a very broad application prospect to improve the growth efficiency and health level of aquatic animals by regulating the structure of intestinal microbiota. The growth-modulating effects of gut microbiota in aquatic species may be mediated through their regulatory roles in host lipid, protein, and carbohydrate metabolism. Dietary modulation of gut microbiota significantly enhanced lipid metabolism in grass carp (*Ctenopharyngodon idella*) [58]. Notably, conventionalized zebrafish (*Danio rerio*) exhibited 2.3-fold greater intestinal lipid accumulation than germ-free counterparts, concomitant with upregulated expression of lipid metabolic regulators *cox15*, *ppary*, and *slc2a1a* [59]. Wu et al. [60] demonstrated that the gut microbiota of *Nile tilapia* exhibited significant positive correlations with intestinal metabolic derivatives, particularly those involved in carbohydrate metabolism pathways. Gastrointestinal microbiota de novo synthesizes essential amino acids to regulate AA homeostasis, while also metabolizing significant amounts of AA/proteins through bacterial and epithelial actions [61]. Gut microbes modulate glucose homeostasis, anti-lipidemic effects and increasing short-chain fatty acids, and increased expressions of genes related to carbohydrate metabolism and innate immunity, along with down-regulation of oxidative stress-related genes, thus affecting host metabolism and growth [62].

The characterization and comparison the intestinal microbiota of *B. areolata* is complex, which the relative abundance of intestinal microbiota was different between the two groups with different growth rates. Compared with SG group, the microbial structure of FG group was more reasonable, which may be one of the reasons affecting its growth. Notably, the limited sample size restricted our capacity to comprehensively assess the intestinal microbial diversity of *B. areolata.* Expanding the sample size would not only enhance our understanding of its microbial communities but also facilitate the identification of taxonomically and functionally significant taxa. This methodological constraint warrants particular attention in future comparative studies. Moreover, it will be important to perform more studies that characterize how differences bacterial populations present in intestinal samples directly affect their growth rate.
